# Crying Babies

**Published:** 1877-08

**Authors:** 


**CRYING BABIES.**—A "Crying-Baby" can be bought any day on Broadway for a "quarter;" many a poor, tired fellow would "clear out" his stock in that line at a much "lower figure," if you could come across him about daylight any morning. With a view to abate this crying nuisance, which exists in so many households, a portion of our book on "SLEEP" is devoted to a detail of a plan by which, in every case, if there is no actual disease, children, even infants, without an atom of physic, can be made to sleep all night habitually, without a single squeak; and how, also, to avoid the "colic," which makes the little responsibilities squirm around and squall away, as if a young boa-constrictor were experimenting on every bone in the body, sending all sleep to the antipodes, and "raising a muss" generally.

